# Predicting outcome and recovery after stroke with lesions extracted from MRI images^☆^

**DOI:** 10.1016/j.nicl.2013.03.005

**Published:** 2013-03-22

**Authors:** Thomas M.H. Hope, Mohamed L. Seghier, Alex P. Leff, Cathy J. Price

**Affiliations:** a Wellcome Trust Centre for Neuroimaging, University College London, UK; b Institute of Cognitive Neuroscience, University College London, UK; c Department of Brain, Repair and Rehabilitation, Institute of Neurology, University College London, UK

**Keywords:** Stroke, Aphasia, Speech production, Recovery, Machine learning, Gaussian processes

## Abstract

Here, we present and validate a method that lets us predict the severity of cognitive impairments after stroke, and the likely course of recovery over time. Our approach employs (a) a database that records the behavioural scores from a large population of patients who have, collectively, incurred a comprehensive range of focal brain lesions, (b) an automated procedure to convert structural brain scans from those patients into three-dimensional images of their lesions, and (c) a system to learn the relationship between patients' lesions, demographics and behavioural capacities at different times post-stroke. Validation against data collected from 270 stroke patients suggests that our first set of variables yielded predictions that match or exceed the predictive power reported in any comparable work in the available literature. Predictions are likely to improve when other determinants of recovery are included in the system. Many behavioural outcomes after stroke could be predicted using the proposed approach.

## Highlights

• We use lesion information to predict speech production skills in 270 stroke patients.
• We validate our approach with both cross-sectional and longitudinal patient data.
• Better predictions employ more relevant and detailed lesion site information.

## 1 Introduction

Stroke can have long term consequences on cognitive and/or sensory-motor functions leaving patients keen to learn whether, when, and in what respects, they might be expected to recover. Most attempts to answer these questions are focused on recovery during the first weeks or months after the insult occurred (Konig et al., 2008; Pedersen et al., 2004; Tilling et al., 2001), the presumption being that meaningful recovery will happen during that time, or not at all (Kotila et al., 1984; Moss and Nicholas, 2006). Increasingly however, evidence has begun to emerge that stroke patients can and do make significant gains even many years after their insult occurred (Moss and Nicholas, 2006; Smania et al., 2010). With increasing evidence that recovery can occur in the long term (Berthier et al., 2011), there is an increasing need for tools and techniques to predict much longer term prognoses for stroke patients.

In what follows, we propose a practical way to start to make these predictions, founded on the intuition that the cognitive and behavioural symptoms a patient suffers, and the likely course of recovery from them, depend on what brain regions were damaged by their stroke (Price et al., 2010a). Following a tradition that dates back to the first attempts to make practical use of Bayesian logic to predict variables of clinical interest (e.g. (Gorry, 1973; Szolovits, 1978)), we employ Gaussian Process model Regression (GPR) to try to learn the relevant structure–function-recovery associations from a large sample of stroke patients, and to use those relationships to predict prognoses for new patients. The result is a ‘recognition model’ (Friston and Ashburner, 2004) for cognitive symptoms and recovery throughout the first decade and more post-stroke, expressed in probabilistic terms. In what follows, we demonstrate and validate the use of the GPR technique for predicting behavioural outcome after stroke. Our outcome of interest was the recovery of speech production over time, as this behaviour is well characterised in the patient data we already have available in our PLORAS database (Price et al., 2010a).

The main purpose of this work is to test the predictions made by an algorithm that uses a combination of lesion and non-lesion factors. In this case, we are less concerned with where the lesion sites are and more concerned with the accuracy of the predictions. This approach differs from that taken by many other types of lesion analysis. Indeed, our goal was to move beyond previous lesion-analysis approaches by: (1) making predicted prognoses at the level of individual patients rather than looking for statistically significant group-level differences that are traditionally implemented with techniques like Voxel-based Lesion Symptom Mapping (VLSM) and Voxel Based Morphometry (VBM); (2) providing continuous predictions over time so that temporal changes in the lesion-symptom mapping can be modelled; (3) considering how combinations of lesion factors influence symptom outcome rather than treating each voxel independently; (4) testing many combinations of lesion factors rather than focusing on a priori regions of interest; (5) employing dimensionality reduction techniques which manipulate variables independently (i.e. damage to different regions, time post-stroke, and other demographic variables) rather than using principle components as in analyses such as Partial Least Squares (PLS) where the source of the variance is more difficult to interpret; (6) using non-linear regression to provide greater flexibility and power to the analysis; and (7) providing probability distributions with each prediction in contrast to other non-linear learners, such as Support Vector Machines (SVMs) or Relevance Vector Machines (RVMs), which make single point predictions.

Overall, our intention was to provide a step towards a system that would enable clinicians to predict outcome in a way that is meaningful and relevant to patients, their carers and therapists. This necessitates an approach that can predict outcome accurately, at the individual level, in a continuous way over time post-stroke, and with a quantification of the precision of the predictions. Our GPR approach has the required characteristics, though we do not claim that it is the only algorithm with the desired qualities. Adaptations of many other statistical machine-learning algorithms could potentially perform reasonably well on the same problem.

## 2 Method

### 2.1 The PLORAS database

Our PLORAS database associates stroke patients, tested at a broad range of times post-stroke (from less than a month to more than 30 years), with demographic data (age when the stroke occurred, handedness pre-stroke, gender, first language, and so on), behavioural test scores from the language battery of the Comprehensive Aphasia Test (CAT; (Swinburn et al., 2004)), and high resolution T1-weighted MRI brain scans (Price et al., 2010a).

### 2.2 Patient selection criteria

We included all available patients with stroke irrespective of the site of the lesion, the presence or absence of aphasia, or any other type of cognitive impairments (e.g. spatial neglect or short term memory problems). Patients were only excluded on the basis of their behaviour if they were unable to consent themselves for the study, showed a lack of understanding on why they were participating in the study or were unable to see or hear the stimuli required to assess their speech production abilities. This relatively unconstrained selection approach ensured that patients differed from one another on a number of dimensions that might be important for characterising variability in recovery rates. We only excluded patients if they:

a) were very young (< 20 years) or very old (> 90 years)
b) were less than 1 month post-stroke at assessment
c) had evidence of other significant neurological conditions (e.g. dementia, multiple sclerosis)
d) did not speak English as a first language
e) showed no visible damage anywhere in the brain, as assessed by a neurologist (APL), using the patients' raw T1-weighted scans
f) had suffered dispersed rather than focal damage. To make this judgement, we employed the lesion identification algorithm described previously (Seghier et al., 2008) to identify the damaged regions in the patients' brains, and excluded patients whose lesions occupied less than 100 contiguous voxels (2 mm × 2 mm × 2 mm) in the brain.

We did not require that the patients we included had speech production impairments, or any other type of aphasia, during the acute stage post-stroke. Self-assessment of speech abilities in the early weeks after stroke indicated that some patients did and some patients didn't think that they had difficulties, but this relies on self-report. Given the nature of stroke and aphasia, it is likely that any patient aphasic at the time they entered our study will have been aphasic at the time of their stroke, but note that this is not a necessary condition for the analysis which is cross sectional and contemporaneous, that is, the behavioural and imaging data were collected at the same time. The inclusion of patients whose lesions may never have caused impaired speech was expected to facilitate the learning of which strokes would and would not cause impaired speech production throughout the whole range of times post-stroke. This selection process left us with data from 270 patients, but with repeated assessments for some patients at different times post-stroke, our dataset included a total of 315 speech production scores.

### 2.3 Behavioural data

The CAT defines behavioural T scores for individual patients in a set of 34 different task dimensions. Many of the dimensions relate directly to language (such as ‘reading words’, or ‘repeating non-words’), while others capture more general cognitive capacities such as semantic memory, which can aid the interpretation of the language scores, (see (Swinburn et al., 2004), for details). Each score defines the ability of the patient relative to a distribution of 60 patients with post-stroke aphasia, indicating how well or badly that patient has performed relative to that distribution (thus aiming to avoid ceiling and floor effects where possible). The threshold of impairment in each dimension is then defined relative to a second population of 27 neurologically normal controls; performance below the threshold implies that the patient would be in the bottom 5% of that normal population. Within each behavioural dimension, lower T scores indicate poorer overall performance.

In the current work, the focus of our interest was speech production, but none of the CAT's dimensions capture that capacity independently, so we constructed the measure we actually used as a composite of T scores (see next section for more details) in relevant CAT dimensions. Our ‘speech production score’ was constructed by combining scores from word and sentence repetition tasks (with heard stimuli), an object naming task (using visually presented pictures), and a picture description task (using pictures of events, requiring sentential utterances in response). After aligning the scores in these 4 dimensions to make their impairment thresholds equal (adding constants to three of the four series of scores), the actual speech production score for a given patient was calculated as the mean of each subject's (i) minimum T score in the tasks involving visual stimuli and (ii) minimum T score in the tasks involving aural stimuli — the intention being to track impairments that cut across the two modalities. Accuracy for predicting the outcome of individual tests was expected to be lower than the composite score because each individual score is determined by many different functions (e.g. picture naming requires good object recognition as well as speech production, while auditory repetition requires good auditory word repetition as well as good speech production). The composite T-scores that we generated were then employed as the target outputs for an induction procedure (see below).

### 2.4 Interpretation of the speech production score

The preference for T-scores over raw scores in the CAT flows from the recognition that its different assessments are not all equally difficult (Swinburn et al., 2004; page 103) — so a direct comparison of the raw scores obtained in those assessments (which are, in any case, rarely normally distributed) would likely be misleading, and a standardised metric is required. The cost of that conversion is a non-linear relationship between the results attached to each patient in each dimension, and their behavioural performance on that task. In the word repetition tasks, for example, each correct answer attracts 2 points, or just 1 if there is a significant pause or self correction; a T-score change of 3 points, from ‘52’ to ‘55’, will be observed in a mildly impaired patient who identifies just 1 more object picture correctly to move from a raw score of ‘26’ to a score of ‘28’, but a more severely impaired patient with a T-score of ‘45’ would need at least 11 extra points on the raw score scale to gain the same improvement on the T-score scale. The T-scores represent the position that a patient would have relative to a population of aphasics — they convey ‘abnormality’, rather than strict behavioural performance itself.

Lower scores in our composite measure, which indicate more severe impairment, are driven by lower T-scores for both auditory and visual stimuli in either word or sentence production tasks; the speech production score is designed to track *bimodal* abnormality — or impairment in speaking tasks irrespective of the sensory modality of the stimuli that drive those tasks. Nevertheless, the composite score is still essentially a T-score, with lower values indicating more severe impairment, and higher values indicating less severe impairment.

### 2.5 MRI data acquisition

Imaging was always conducted within 2 weeks of the collection of the associated behavioural data. Scans were acquired either with a Siemens 1.5 T Sonata scanner, or with a Siemens 3 T Trio scanner (Siemens Medical Systems, Erlangen, Germany). In each case a T1 weighted 3D modified driven equilibrium Fourier transform sequence (Deichmann et al., 2004) was used to acquire 176 contiguous sagittal slices with an image matrix of 256 × 224 yielding a final resolution of 1 mm^3^: repetition time/echo time/inversion time = 12.24/3.56/530 ms and 7.92/2.48/910 ms at 1.5 T and 3 T respectively.

### 2.6 MRI pre-processing

The pre-processing stage for these MRI data is identical to that described in Price et al. (2010b). Structural images were pre-processed with Statistical Parametric Mapping software (Wellcome Trust Centre for Neuroimaging, 2005). The images were spatially normalised into standard Montreal Neurological Institute (MNI) space using a unified segmentation algorithm (Ashburner and Friston, 2005) optimized for use in patients with focal brain lesions (Seghier et al., 2008). More specifically, the lesion of each patient was automatically identified using a modified unified segmentation and an outlier detection algorithm (see procedure in Seghier et al. (2008)). The output of the process is a *lesion image* for each patient, in MNI space with a voxel size of 2 × 2 × 2mm. Each voxel codes the degree of abnormality of the underlying tissue (scaled to the range 0 to 1). This *lesion image* is then thresholded to generate a *binary lesion image*, with voxels assigned to the lesion if they composed a group of at least 100 voxels, all of which had abnormality degrees more than 0.3 (see (Seghier et al., 2008)) for a more detailed explanation. These binary lesion images were used to create the lesion overlap map displayed in Fig. 2 and to provide an estimate of lesion size in terms of the total number of lesioned voxels.

### 2.7 Learning

We employed GPR to learn the associations between predictors (patient demographic and lesion data), and output (speech production scores), and how these associations vary with time post-stroke. GPR, described in detail in Rasmussen and Williams (2006), has been successfully applied to a large variety of statistical learning problems in the past, from biomarker discovery (Chu, 2005) and the interpretation of neural spike train data (Eichhorn et al., 2004), to autonomous flight control (Ko et al., 2007) and telecommunications network management (Duel, 1995). Many alternatives to GPR might be just effective in this case, but GPR has two features that make it particularly appropriate to the problem at hand. First, the regression process fits functions of the form:

$$y=f\left(x\right)+\in \left(x\right)$$

where *x* are the predictors, *y* is the target or output variable, and ∈(*x*) is a Gaussian noise term. By attempting to parameterise the influence of the noise on the relationship between the underlying lesion-symptom–time associations, and the behavioural data, the GPR approach should be less susceptible to over-fitting than many of its counterparts in the machine learning literature. Several alternative approaches have been proposed to help minimise over-fitting in the past — such as the attempt, in Support Vector Machine (SVM) classification, to find the hyperplane which maximises the margin, or separation, between classes (Vapnik, 1999). We prefer the approach taken by GPR because it is more transparently interpretable than are most of its counterparts' approaches, while being at least as effective.

The second desirable feature of the GPR framework is that it assigns predictive weights across the whole of its parameter space — representing the likelihood of the observed data given the multiple different parameter values across their range for each regressor and the full combination of regressors — rather than simply trying to fit the single ‘best’ value for each parameter (i.e. those which minimise prediction error overall). The result is a predictive (Gaussian) distribution rather than a single-point prediction; if the distribution has small variance, the implication is that a comparatively small subset of possible parameters makes the observed data much more likely than any other set. In cases like this, we can be more confident that the chosen parameter set is ‘right’ (or close to right) — in other words, the variance of the predictive distribution provides a reasonable measure of the confidence with which particular predictions are made. This association between point-predictions (of continuous variables) and probability distributions seems a natural fit to many prediction problems of clinical interest.

Our implementation of the process was adapted from the NetLab library (Nabney, 2001), employing scaled conjugate gradient optimisation, and a rational quadratic covariance function. The outputs of the process – the predicted prognoses themselves (speech production ability through time, in our case) – are Gaussian probability distributions through time, with the peak/mean function representing the system's mean prediction. When we refer to the variance explained by predictions of a target variable, we have calculated those measures from the mean prediction of the system under study.

### 2.8 Validation

We used leave-one-out cross-validation to assess the learning system's performance with different sets of predictors, employing both a ‘cross-sectional’ configuration and a ‘longitudinal configuration’. In both configurations, we divided the dataset into training and test sets, the goal being to assess how well our Gaussian process models could predict the scores assigned to the patients in the test set, after learning from the patients in the training set. In the cross-sectional configuration, the details from all but one patient are used to predict the speech production score given the lesion information and time post-stroke from the excluded patient. No information about the excluded patient is presented to the learner during training. In the longitudinal configuration, the challenge is the same, but in this case the patient's scan, time post-stroke and behaviour score at an earlier time are presented to the learner during training. The learner then predicts what the future scores for this patient would be. In both cases, the learner is asked to use what it has learned during training to make inferences about new/future data.

In the cross-sectional configuration, we ran 270 of these assessments – one for each patient – in which the training set included all of the 269 other patients in the dataset, and the goal was to predict, on the basis of the test patient's lesion and other predictors, the speech production score actually assigned to them. In the longitudinal configuration, we trained a Gaussian process learner with all of the data from the cross-sectional configuration (i.e. 270 records), then tried to predict the scores assigned at repeat visits to the 38 patients who were assessed more than once.

### 2.9 Experimental design

We conducted a series of linked experiments in which ever-more detailed information concerning the patients' lesions was progressively introduced to drive predictions concerning the way their speech production skills might evolve through time. This design was employed to illustrate (a) how ever-more effective predictions might be made by systematically exploring the space of potential predictors, and (b) that lesion site information can drive those effective predictions. We then consider prediction for the longitudinal data separately. In each case, we report the results of a linear regression of predicted vs. actual speech production scores for each of the patients in our data, as a measure of how well the predictions capture those actual scores. Both learning and validation steps were carried out with scripts written in Matlab (The MathWorks, Natick, MA, USA).

## 3 Results

**Fig. 1:**
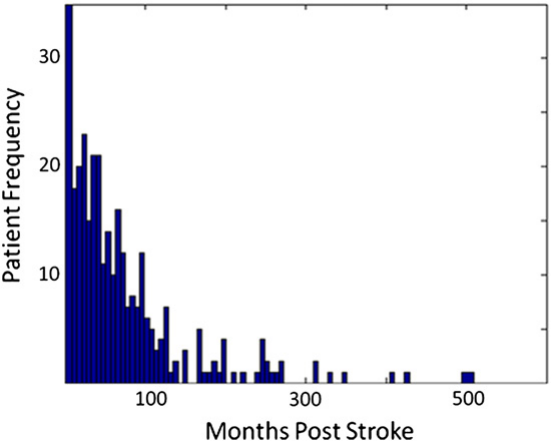
Histogram of the times post-stroke at which patients were assessed. Patient frequencies are calculated in bins of 6 months.

**Fig. 2:**
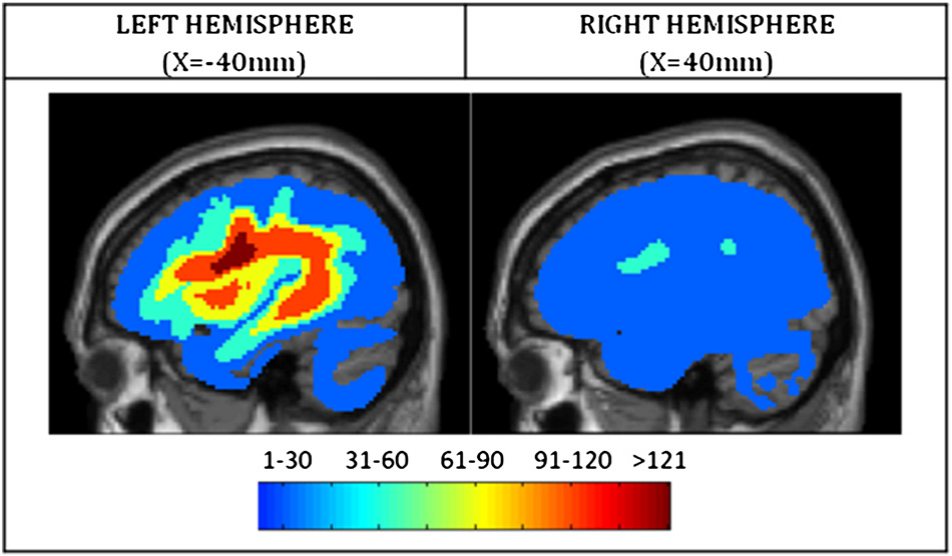
Lesion overlap map showing the frequency of lesions in each hemisphere. The colour in the image indicates the number of patients who had a lesion at each brain voxel. The lesions were identified, in MNI space, using the procedures described in the Method section. After computing the degree of abnormality at each voxel (scaled to the range 0 to 1), a binary lesion image was created by thresholding the degree of abnormality at a cut-off threshold of 0.3 (see (Seghier et al., 2008)). The maximum number of patients whose lesions converged on a single voxel was 152.

Our patients' ages varied from 20 to 90 years old (mean = 61.27; SD = 12.21); see Fig. 1. Our sample included 93 women and 177 men, and 242 of the patients were right handed. The size, location and hemisphere of the lesions varied widely, but most patients had damage in the left hemisphere of the brain; just 50 of the 270 patients had damage restricted to the right hemisphere (as defined by our lesion identification algorithm, described previously (Seghier et al., 2008)). Fig. 2 illustrates the lesion overlap map for the selected patients, and the sampling of lesions in the left and right hemispheres.

### 3.1 Learning without lesion information

Our first and simplest experiment evaluated the predictive value of:

1. the time elapsed since the stroke occurred;
2. their age when the stroke occurred;
3. their handedness before the stroke occurred; and
4. their gender.

This information has previously been claimed to be predictive of aphasic stroke patients' initial symptoms and eventual prognosis, but these claims are disputed (Plowman et al., 2011). Our results add to that doubt, yielding predictions that do not appear to capture any significant proportion of the variance of those scores (R^2^ = 0.01, F = 2.22, p = 0.14). This first and non-significant result provides a useful control for the subsequent analyses because it allows us to illustrate the benefit that lesion information can bring to the prediction problem after showing what is explained (or not explained) when no lesion information is used.

### 3.2 Learning with lesion volume

The poor predictive performance reported so far was expected because the predictions took no account at all of the patients' lesions. Our next experiment sought to begin to redress that imbalance by including lesion volume (expressed as the total number of lesioned voxels) — information that has been recognised as predictive of aphasics stroke patients' initial symptoms and eventual prognosis (Plowman et al., 2011). As expected, access to this kind of information (alongside the demographic predictors used previously) did improve the predictions (R^2^ = 0.35, F = 144.73, p < 0.001), and also significantly reduced prediction error relative to learning without any lesion information at all (Wilcoxon test: Z = 6.64, p < 0.001).

### 3.3 Learning with lesion site 1: lateralised lesion volume

Our next experiments all sought to add lesion site information to the predictors. Our first attempt was deliberately minimal, focussing on lateralised lesion volume — or the number of lesioned voxels in each of the left and right hemispheres of the brain. Access to this kind of information, in addition to lesion volume and the non-lesion data used so far, increased the strength of the statistical relationship between actual and predicted speech production scores (R^2^ = 0.47, F = 239.36, p < 0.001), significantly reducing prediction error relative to learning with lesion volume and demographics alone (Wilcoxon test: Z = 4.25, p < 0.001).

### 3.4 Learning with lesion site 2: atlas-based lesion coding

Next, we sought to measure whether, and to what extent, finer-grained lesion-location data might improve the predictions still further. We derived that information by encoding each patient's lesion as the proportions of anatomically defined regions of interest that the lesion appears to destroy. The cortical regions of interest were extracted from the Anatomy Toolbox (Eickhoff et al., 2005), and the white matter tracts of interest were extracted from the ICBM-DTI-81 white-matter label atlas (Mori et al., 2006) and the JHU white-matter tractography atlas (Hua, 2008). There were 232 regions in all, so each patient was associated with 232 ‘lesion site’ predictors, varying in the range 0–1. This move to atlas-based lesion coding is effectively a kind of dimensionality reduction — the benefit being that the resultant predictors might be more interpretable than those extracted using more traditional numerical methods. By replacing the 2 lateralised lesion volume predictors with these 232 new atlas-based predictors, we improved the predictions again (R^2^ = 0.52, F = 294.24, p < 0.001), although the reduction in prediction error was not significant (Wilcoxon test: Z = 1.34, p = 0.18).

### 3.5 Learning with lesion site 3: atlas-based lesion features

It would be very surprising if every one of those 232 regions was equally relevant to the implementation or recovery of speech production skills. The implication is that many or most of our atlas-based predictors are irrelevant to the task at hand, and to the extent that this is true, random correlations between those irrelevant predictors and the target measure would be expected to hamper effective predictions (Oomen et al., 2008), masking the benefits of including higher resolution lesion site information. By throwing those less relevant predictors away, we expected to see at least some improvement in the overall performance of the system — as well as a significant reduction in prediction error relative to learning with lateralised lesion volume (as in Section 3.4).

**Fig. 3:**
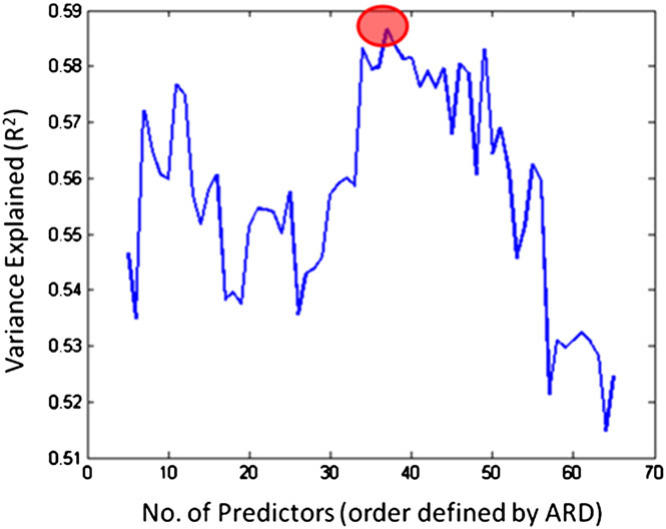
Variance explained by predictor subsets. Variance explained when predictor subsets are used to make predictions for every patient with a leave-one-out cross-validation procedure. Starting with the 5 most relevant predictors (where relevance was defined via ARD), each successive subset is constructed by adding the next most relevant predictor to those already considered (up to a maximum of 65). As expected, the most effective subsets tend to include fewer predictors, though predictive performance drops off when fewer than 10 predictors are used. The maximum point on this graph yields out preferred predictor configuration, but many other subsets can be used to drive predictions which capture almost as much of the variance in the speech production scores actually assigned to the patients when they were assessed.

To select relevant lesion features, we used Automatic Relevance Determination (ARD) — a Bayesian filter method driven by an initial pass of Gaussian process model learning across the whole of the dataset, in which individual hyperparameters are learned for each predictor (Mackay, 1992; Neal, 1996). Our implementation of the approach is adapted from the NetLab software package (Nabney, 2001). Armed with this reduced set of scores, we re-ran the validation multiple times with increasingly large subsets of the predictors, focussing on those judged most relevant by ARD (i.e. growing the predictor configuration in the order defined by their relative relevance scores). Fig. 3 displays the variance explained by the subsets containing the first 5–65 of the predictors selected by ARD. The best subset included 37 relevant predictors (R^2^ = 0.59, F = 38.38, p < 0.001), including time post-stroke (the most significant, single predictor), lesion volume, and encroachment into 35 regions of the brain (listed in Table 1). Prediction error in this configuration was significantly reduced both relative to learning with the full set of (232) atlas-based predictors (Wilcoxon test: Z = 2.12, p = 0.034), and relative to learning with lateralised lesion volume alone (Z = 2.96, p = 0.003).

### 3.6 Summary of cross-validation results

A summary of the validation results is detailed in Table 2 for the composite measure of speech production and for each speech production test individually. As expected, the predictive performance for the composite speech production measure is better than that for any of the individual test scores which were used to calculate the composite. The best feature subset that we found for our speech production score generates predictions with significantly less error than do the best feature subsets for any of the 4 components of that speech production score (see Table 3).

### 3.7 Predicting longitudinal data

As determined by ARD, our preferred predictor configuration for speech production skills is a combination of time post-stroke and lesion damage. To explore the influence of time directly (i.e. within-patient, rather than across patients), we used the learner to predict what will happen to particular patients over time. 38 of the 270 patients in our original dataset were assessed more than once, but only the *first* of those assessments was used in the cross-sectional analysis. In this section on the longitudinal data, we validate our proposed approach by attempting to predict the speech production scores assigned to those 38 patients *after* that initial assessment. After learning from just the patient data used for the cross-sectional configuration, our learner can predict the later assessment scores very accurately (R^2^ = 0.84, F = 225.00, p < 0.001; see Fig. 5).

### 3.8 Examples of predicted prognoses

In Fig. 6, we display examples of prognoses predicted for two different patients, employing our preferred predictor configuration (as detailed previously). In each case, the predictions were made after training with data from every other patient in our dataset (but without reference to the ‘test’ patient's own data). The prognoses themselves are then derived by using our preferred predictor configuration to predict the test patient's speech production scores at every month post-stroke.

## 4 Discussion

Using minimal demographic data, together with lesion data extracted from MRI images, we have demonstrated that stroke patients' speech production skills can be predicted, with reasonable accuracy, throughout the first decade and more after stroke. The lesions suffered by the members of our aphasic patient population are very variable, so their predicted prognoses are very variable too, but our results suggest that, on average, this population might be expected to continue to recover for many years post-stroke. This is consistent with emerging evidence that aphasic stroke patients can and do recover over much longer timescales than previously thought (Maas et al., 2010; Moss and Nicholas, 2006; Smania et al., 2010).

The comparison between this work and its precedents in the literature is complicated by several factors. First, the focus of most of those prior studies of stroke recovery is on the first few days, weeks, or months post-stroke (Counsell, 2002; Ferro et al., 1999; Konig et al., 2008; Laska et al., 2001; Lazar and Antoniello, 2008; Mazzoni et al., 1992; Tilling et al., 2001), following the presumption that meaningful recovery occurs quickly, or not at all (Ahlosio et al., 1984; Knopman et al., 1983; Moss and Nicholas, 2006; Silbeck et al., 1983). To our knowledge, the current work is the first reported attempt to predict prognoses over such a long period post-stroke.

Second, many potentially comparable studies employ bespoke outcome variables (see (Veerbeek, 2011), for a review), reflecting the particular demands of their proposed clinical context. The overwhelming emphasis on prognoses during the acute and/or sub-acute stages post-stroke naturally lends itself to a focus on variables of particular and immediate clinical interest, such as the risk of death (Wiberg, 2012), the likelihood that a patient can be discharged (Jørgensen, 1997), or the patient's short-term ability to perform basic, daily tasks (Veerbeek, 2011). Our own results illustrate the sensitivity of predictive methods to the particulars of the outcome variable employed (see Table 2 and Table 3); it is no surprise that, given a defined set of predictors, some outcome variables are simply more ‘predictable’ than others. That variability encourages caution when comparing methods across different outcome variables.

Finally, and perhaps again encouraged by their clinical context and preferred outcome variables, most prior studies cast the prognosis-prediction problem in terms of classification (e.g. into ‘impaired’ and ‘unimpaired’ groups), rather than in terms of the regression of a continuous variable through time (Ahlosio et al., 1984; Allen, 1984; Counsell, 2002; Perdersen et al., 1995). This is often true even when the underlying variable of interest is continuous, thereby necessitating the definition of a ‘cut-off’ score (Veerbeek, 2011). Our own predictions can be expressed in these terms by imposing a cut-off score of 60 on the speech production scores — patients with scores below this threshold being within the bottom 5% of the range expected of neurologically normal controls. By varying the decision threshold imposed on the predictions, we can approximate a Receiver Operating Characteristic curve from our predictions, with an area under the curve of 0.84 (95% CI: 0.80–0.89) for the cross-sectional configuration, and 0.96 (95% CI: 0.85–1) for the longitudinal configuration. But we suggest that this binary treatment of the problem is inappropriate to the problem at hand, both because the cut-off is largely arbitrary, and because a binary treatment ignores the improvement that patients might make within the ‘impaired range’ — improvement that could have a significant impact on their eventual quality of life.

While bearing these complicating factors in mind, it should be noted that the best comparisons we can make with past work appear to be favourable. Perhaps the most naturally comparable work in the stroke prognosis prediction literature is that by Tilling et al. (2001), who used multi-level growth curve models to predict continuous trajectories, on a 20-point Barthel Index, for patients throughout the first few weeks and months post-stroke. Unlike the work reported here, these authors only employed quite coarse-grained lesion data in their analysis (classifying lesions as ‘ischemic’, ‘hemorrhagic’, or ‘ill defined/unclassified’). But like us, Tilling and colleagues report two configurations for their inducer: a ‘cross-sectional configuration’, in which predictions are made without knowledge of the test patients' past behaviour scores, and a ‘longitudinal configuration’, in which that past behaviour is known. In each case, their predictions are assessed by measuring the standard deviation of the error distribution — the idea being that narrower prediction error distributions imply minimal prediction error overall (assuming that the mean is close to zero). In their cross-sectional configuration, the standard deviation of the prediction error distribution is 5.43, while in the longitudinal configuration, it is 3.84; their patients' scores ranged between ‘6’ and ‘20’ on the Barthel scale, so these standard deviations correspond to 36.2% and 25.6% of that range respectively. By contrast, our cross-sectional and longitudinal configurations yielded error distribution standard deviations of 5.43 and 3.58 respectively — or 17.8% and 11.7% respectively of the range for our speech production score (35.5–60). In other words, while bearing the preceding caveats in mind, and recognising that Tilling and colleagues employed much larger validation sets, our predictions compare favourably with theirs.

Our results also compare favourably with a recent attempt to use a method more closely analogous to our own – the Relevance Vector Machine (Tipping, 2000) – to predict scores on standard behavioural assessments for Alzheimer's disease from subjects' MRI images (Stonnington et al., 2010). In their favoured configuration, these authors report an R value (correlation coefficient) of 0.73 for the relationship between predicted and actual scores. This corresponds to an R^2^ of 0.53 — roughly equivalent to the predictive power that we could achieve with the complete set of 232 atlas-based predictors (i.e. before deploying feature selection). Gaussian Process regression has yielded much stronger predictions of subjects' ages from their MRI scans in the past (R^2^ = 0.92, mean error = 4.98 years; see (Ziegler and Gaser, 2012)), which is itself relevant to the diagnosis of Alzheimer's, but no attempt was being made to predict behavioural performance in that case. In other words, our predictions for behaviour after stroke appear to be powerful relative to analogous past work.

### 4.1 Key predictors

Our approach allows us to combine the influence of multiple different predictors to provide a more accurate prognosis for recovery. In the example given, the preferred predictor configuration for our speech production measure included time post-stroke, and encroachment of the patient's lesion into 35 regions of the brain (list in Table 1). The influence of lesion site information on speech production abilities is consistent with the conclusions of the recent review by Plowman et al. (2011). The specific lesion sites identified in Table 1 also appear to be consistent with previous studies. Alongside Wernicke's area, which was naturally expected to appear in our preferred predictor configuration, many of the grey matter regions most emphasised have also been associated with speech production skills in the past: the parietal operculum and primary auditory cortices in sensory monitoring (Simmonds et al., 2011); the hippocampus with word retrieval in production tasks (Hocking et al., 2009; Whitney, 2009); the insula with articulatory coding/motor programming (Petersen, 1989); the somatosensory cortex with sensory feedback during speech (Bohland, 2006); the cerebellar regions with vocalisation and breathing during articulation (Nota, 2004), and with the rate of articulation (Frings, 2006); the inferior parietal lobule with speech repetition (Fridriksson, 2010); and the thalamus with extensive influence in the speech network (Johnson and Ojemann, 2000).

The presence of so many white matter tracts in our preferred configuration is consistent with the increasing emphasis on these tracts in anatomical models of language and cognition (e.g. (Bernal and Ardila, 2009)). Following a recent reanalysis of the patients examined by Broca (Dronkers et al., 2007), for example, the potentially surprising absence of Broca's area in Table 1 might best be explained as consistent with the claim that the white matter underlying that area plays a rather more important role in the speech production network than the grey matter itself.

But though many of the regions selected appear to make sense given the context, the temptation to over-interpret them should be resisted because their roles as predictors remain unclear. In Fig. 3, we can see that similar levels of predictive power can be achieved using fewer than half of the predictors that compose our preferred configuration. The implication is that many of these predictors encode redundant or strongly correlated information. In that context, it may be too early to try to draw very firm conclusions from the atlas regions that compose our preferred predictor configuration — more data would seem to be required before we can expect to use ARD to properly tease apart the influence of these different regions.

This conclusion is supported by the recognition that, large though it is, our own dataset is still skewed in some important respects. For example, only 50 of the 270 patients had lesion damage restricted to the right-hemisphere alone; 34 of these had speech scores in the normal range and the remaining 16 had mildly aphasic scores (the minimum score in this group was ‘54’) with only one right-hemisphere region contributing to our list of preferred predictors (see Table 1). Skews like this should encourage caution when interpreting the regions that drive our predictions.

### 4.2 Limitations of the current study

One criticism of the system described here is that it pools scans taken both soon after stroke (within the first month), and many years later (30 years or more). Since the size and site of a patient's apparent lesion(s) may vary post-stroke, this approach could introduce noise into the structure–function-recovery relationships that we have attempted to learn, thus reducing the sensitivity and accuracy of our predictions. One way to reduce that noise would be to restrict our dataset to patients either during the acute/sub-acute phase post-stroke (Karnath et al., 2011), or to patients who are well into the chronic phase (Price et al., 2010b). But this kind of restriction will also naturally reduce the size of our dataset, and perhaps as a consequence, we found no evidence that we could improve our predictions this way.

**Fig. 4:**
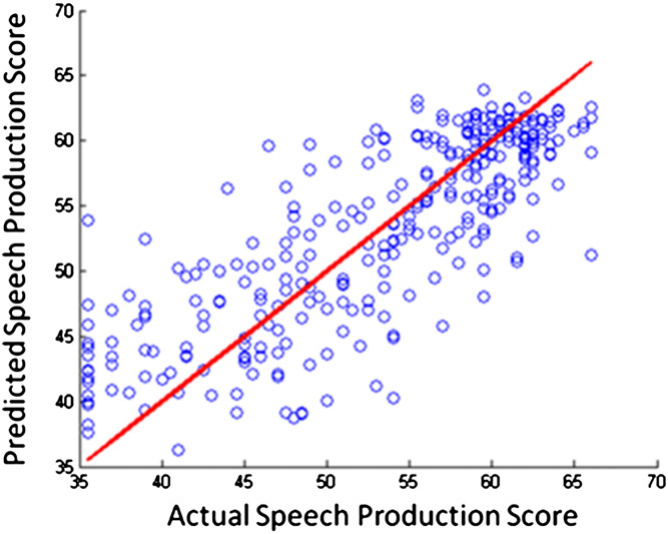
Actual vs. predicted speech production scores. Predicted speech production scores plotted against the actual scores assigned to the patients in our dataset. Predicted scores would be equal to actual scores if they fell along the red line.

Another more immediate criticism is that our preferred predictor configuration systematically under-estimates the severity of the impairments suffered by the most impaired patients in our dataset (i.e. by predicting speech production scores consistently above those actually assigned on assessment; see Fig. 4). This may be an artefact of the measures on which our speech production score was based since, at poorer levels of performance, small differences in the underlying raw scores from the repeating and naming tasks yield larger differences in the T-scores calculated from them. Further analysis will be needed to determine if different measures of speech production skills can mitigate this skew.

Another concern is that new patients might exhibit relationships that have simply never appeared in our data so far. Since the relationships that we learn between lesions, symptoms, and time are learned solely from the data we have, predictions made for these ‘novel’ types of patients are more likely be wrong (see (Stonnington et al., 2010), for a discussion of this). This concern is mitigated by the large sample of patients employed here, and it will be mitigated further as our sample of patient data increases — as it will do, dramatically, during the next few years. This change should also improve the accuracy and reliability of the predictions that we can make for the current sample of patients. However, in its current form, our preferred predictor configuration clearly makes rather coarse distinctions between patients with right-hemisphere damage only (since only 1 of the regions in our preferred configuration is in the right hemisphere of the brain). As the representation of such patients increases in our dataset, so too should the representation of right hemisphere regions in our list of preferred predictors.

**Fig. 5:**
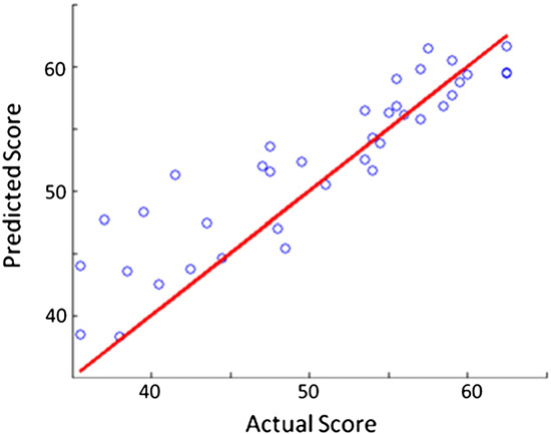
Actual vs. predicted speech production scores for longitudinal data.

**Fig. 6:**
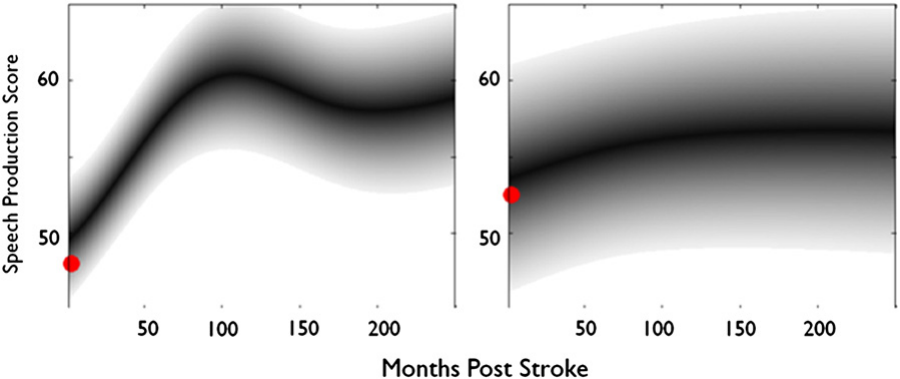
Two examples of predicted prognoses. Two predicted prognoses for different patients in the database. Each prediction was made after removing the ‘test patient’ from the data, and then training with the rest; the prognoses are then constructed by making 240 individual predictions for the test patient, by replacing their ‘time post-stroke’ value first with ‘1’, then with ‘2’, and so on up to ‘250’. In each plot, the actual data measured at assessment is plotted in red (both of these patients were assessed within a year after their stroke occurred). Both predictions are expressed as probability distributions through time, with the mean prediction in black, and borders at 2 standard deviations from that mean prediction (i.e. 95% confidence). (Left) A prognosis which captures the patient's real score with reasonable accuracy (i.e. the mean prediction is close to the real score), and which predicts, with reasonably high confidence, that the patient will improve throughout the first 100 months post-stroke, before reaching a plateau somewhere around the impairment threshold (score = 60); the confidence attached to the prediction is comparatively high initially, but decreases after about 100 months, as indicated by the increase in the variance of the predictive distribution after that time. (Right) A prognosis predicting comparatively little improvement over time, which captures another patient's actual data quite accurately, but which is made with comparatively low confidence.

Results of this sort will always benefit from further validation with independent samples of data. In this work, we have selected a preferred configuration on the basis of its performance within and across the data we have, which raises the spectre of over-fitting in the feature selection process, and consequent questions concerning its generalisation to independent data. Studies of this problem have suggested that it may be comparatively minor in datasets which, like ours, contain more than 250 items (Kohavi, 1997). And in our case, the concern is further mitigated by the recognition that the quality of our predictions does not dramatically decline when we exclude many of the predictors in our preferred configuration (see Fig. 3), and by the fact that the system performs so well on longitudinal data (see Fig. 5).

Finally, it should be mentioned that statistical machine learning approaches, like GPR, SVM, RVM, and many others, are all susceptible to criticism because they lack the kind of transparency that might be expected of more conventional (typically linear) regression models. Our experience is that the predictive power reported here simply is not attainable, in this domain, with these more transparent techniques; we have sought to maximise that power here, which has encouraged the use of a more complex machine learning approach. This choice trades power for transparency, because for most practical purposes, GPR – like SVM, RVM, and a host of other alternatives – is a ‘black box’ system. If another, more transparent method can be found which matches or exceeds the performance that we can get from these more complex methods, it would likely be preferred. However, at present, we take the view that the gains in predictive power attendant on the use of GPR or other similar nonlinear techniques justify the cost in terms of transparency.

### 4.3 Proposed extensions

The approach reported here could naturally be extended in a variety of ways, and there is every reason to hope that our predictions could be further improved by any or all of them. First, the apparent success of our feature selection approach suggests that further gains might be made by exploring other, more rigorous alternatives to it. Though much more computationally expensive than ‘filter’ feature selection methods like ARD, ‘wrapper’ feature selection methods such as backward selection are generally thought to yield better results (Kohavi, 1997). Improved performance might also flow from the addition of different predictors conveying information not considered here — like initial stroke severity and acute behavioural symptoms (Laska et al., 2001; Pedersen et al., 2004), or type and dose of therapy received.

Other promising extensions of this work involve either adding predictors calculated from different sources of data, or using prior knowledge of, or results concerning, relevant structure–function associations to create custom predictors. In recent work, for example, Saur and colleagues have illustrated that functional imaging data might be made to play an analogous role to the structural brain images employed here (Saur et al., 2010) — driving predictions concerning the patient's progress in the future. This study only made predictions a few months ahead, but there is every reason to allow that this kind of approach – perhaps in concert with that reported here – might work on longer timescales too. Similarly, Diffusion Tensor Imaging (DTI) studies have increasingly begun to highlight the role of tracts and tract disruption in the complex behavioural symptoms associated with focal brain lesions (Bernal and Ardila, 2009) — consistent with the emphasis our own results place on white matter. To the extent that DTI can reveal new information that predicts symptoms and recovery post-stroke, an approach along the lines described here should be capable of employing it. Indeed, where we know that damage to particular regions or tracts is relevant to the impairment of interest (Marchina et al., 2011; Price et al., 2010b), it would likely be desirable to design custom predictors to reflect that knowledge — building the fruits of our continuing study of the brain into a tool that can exploit, and test, that knowledge directly.

Anatomical models of speech and language could also be used to guide predictions for recovery, and conversely the lesion sites associated with speech and language difficulties could be used to modify and update anatomical models of speech. At present, however, the interpretation of the current results, in terms of anatomical models of speech, is difficult — because many different combinations of our ‘region predictors’ can generate predictions which are only slightly less good than those which we report. This might mean that, despite including data from 270 patients, we need more patients before we can delineate fine grained descriptions of all the lesions that cause aphasic speech. This is why, in the current work, we prefer to interpret the feature selection results simply as an illustration that better predictions can be made by selecting better, more relevant lesion features. Future work will hopefully be able to marry model-drive and data-driven approaches. However, this is likely to require a larger dataset and/or a very refined set of anatomically defined regions.

Finally, the results reported here indicate that our speech production score was more ‘predictable’ than any of its components (see Table 3). That result naturally encourages a broader search for behavioural measures which might be more or less predictable given the information that we have, or can gather, about a given patient. While working to improve the predictions made so far, we will also begin to expand their scope to include many of the other cognitive capacities that can be impaired by stroke — aiming to provide stroke survivors with prognoses that are as complete and comprehensive as we can make them.

## 5 Conclusion

Stroke patients want to know whether, when, and in what respects, they might expect to recover. We have described the practical basis of a framework that could be used to answer those questions, and illustrated that reasonably accurate predictions can be made given knowledge of (a) the time since the patient's stroke occurred, (b) the volume of the patient's lesion, and (c) detailed information concerning the brain regions damaged by that lesion. We expect to be able to improve on these predictions by refining and extending the data we use to drive them — aiming to build a tool that can eventually deliver predicted prognoses to stroke patients and their carers. For some, the road to recovery may be much longer than they'd hoped, but for many, the knowledge of what they might recover – even in the long term – could be invaluable.

## Figures and Tables

**Table 1:** Regions employed by our preferred predictor configuration to encode lesions.

Grey matter (left hemisphere unless specified)	White matter (left hemisphere unless specified)
• Inferior parietal lobule (PFop, PFm, PFcm, PFt) • Hippocampus (cornu ammonis) • Intraparietal sulcus (IP1) • Insula (Ig2) • Parietal operculum (OP1) • Thalamus (temporal) • Auditory cortex (TE12) • Wernicke's area (TE30) • Somatosensory cortex (1, 2, 3b) • Premotor area (6) • Primary motor cortex (4p) • Cerebellum (IX) — right hemisphere	• Uncinate fasciculus • Fronto-occipital fasciculus • Superior longitudinal fasciculus • Inferior longitudinal fasciculus • Cingulum of the hippocampus • Fibres connecting: o the superior, middle and inferior frontal gyri o the corpus callosum o the precentral and postcentral gyri o the inferior and middle occipital gyri o the middle and superior temporal gyri o the thalamus o the superior parietal lobule

**Table 2:** Summary of cross-validation results for the different predictor configurations.

Predictor configurations	Variance explained (R^2^) of behaviour scores by predictions				
	Speech prod.	Rep. sent.	Obj. name	Rep. wd	Pic. desc.
Demographics only (DEM)	0.01	0.01	0.01	0.04	0.00
DEM + lesion volume (LV)	0.35⁎	0.24⁎	0.26⁎	0.29⁎	0.33⁎
DEM + LV + lateralised lesion volume	0.47⁎	0.38⁎	0.37⁎	0.29⁎	0.40⁎
DEM + LV + atlas-based lesion data (ALD)	0.52⁎	0.42⁎	0.43⁎	0.28⁎	0.42⁎
DEM + LV + ALD features (best)	0.59⁎	0.49⁎	0.51⁎	0.34⁎	0.50⁎

⁎ p < 0.001.

**Table 3:** Comparing the predictive power for CAT component scores to that achieved for the aggregate speech production score (using the Wilcoxon test).

Repeating sentences		Repeating words		Naming objects		Describing pictures	
Z	p	Z	p	Z	p	Z	p
9.11	< 0.001	5.85	< 0.001	2.46	0.014	2.96	0.003
